# 

**Published:** 1883-05

**Authors:** 


					﻿Whole Number,
/CCCCLX.
May, 1883.
Number 5,
Vol. XLVI.
THE
CHICAGO
MEDICAL
Journals Examiner.
CHICAGO:
PUBLISHED BY THE CHICAGO MEDICAL PRESS ASSOCIATION
188 Clark Street.
g4S~Wead the Notice of this Journal among Advertisements.
				

